# Comparison of bird assemblage structures and diversity patterns between seasons among two Ethiopian wetlands

**DOI:** 10.1186/s40850-023-00164-x

**Published:** 2023-03-11

**Authors:** Numeri Awash, Wondimagegnehu Tekalign

**Affiliations:** Wolaita Sodo University, Natural and Computational Sciences College, Biology Department, PO Box 138, Wolaita Sodo, Ethiopia

**Keywords:** Bird species, Relative abundance, Species diversity, Species similarity

## Abstract

Wetlands are significant habitats for bird populations, and knowledge of the diversity and other ecological aspects of bird species contribute to the management of the ecosystem. The present study was based on comparative studies of the diversity and relative abundance of bird species in the two wetlands of southwest Ethiopia. The point count method was utilized in this study. For the data analysis, the Shannon–Weaver diversity index, independent sample t-test, and similarity index were employed. A total of 36 bird species under 11 orders and 24 families were identified. The species diversity and relative abundance were higher in both wetlands during the wet season. The Loga wetland had the higher diversity (H’ = 3.089), whereas the lowest species diversity (H’ = 2.643) was recorded in the wetland of Hurri. During the dry season, the Loga wetland (H’ = 2.738) and the Hurri habitat (H’ = 2.283) had higher and lower diversity, respectively. Seasonal variations in bird species diversity are not statistically significant (*p* > 0.05). Although the two wetlands support several water birds, they have received no conservation attention from concerned bodies. Further follow-up studies over a long period will help determine species-specific conservation measures for wetland-dependent birds.

## Introduction

Wetlands are among the most productive ecosystems in the world, rich in biodiversity and harboring many globally threatened species [1]. These areas play critical ecosystem roles such as biodiversity conservation, hydrological balance, and human welfare [2]. A wide variety of birds use wetland habitats for all or part of their lives [3]. Wetland birds are extremely diverse, reflecting early anatomical and physiological adaptation to this unique but rich habitat [4]. There are two categories of wetland birds: wetland specialists and generalists. Wetland specialists are birds that are entirely dependent on aquatic habitats and cannot survive in any other environment. Generalists, on the other hand, are birds that visit and rely on wetland habitats for food, shelter, and perching [5].

Wetland birds are an important component of the biotic community in an ecosystem [6]. They are good indicators of terrestrial and aquatic ecosystem pollution [7]. They differ widely in their species composition and relative abundance within a community [4]. Bird species diversity is a function of the number of species present and the evenness with which the individuals are dispersed among these species [8, 9].

Elucidating the patterns of species diversity and their abundance across different locations is a vital purpose of community ecology. Many scholars studying species diversity [10] have given emphasis to the bird communities. One of the main priorities in animal conservation is checking their populations to find the best strategies for their sustainable survival [7].

Ethiopia has 18,587 km^2^ of wetlands, though their resources are not entirely known. This is equivalent to about 1.5% of the country's total area [11, 12]. Mengistu described 245 bird species in Ethiopia. Despite the rich bird species in Ethiopia, due to enormous habitat degradation, fragmentation, and loss, the survival of many bird species, including wetland birds [13], along with different types of agroforestry systems, is threatened [14]. In Ethiopia, the wetlands are frequently considered wastelands and are believed to pose obstacles to farming expansion, cause an increase in risks to human and animal health, and be associated with disasters such as floods, with consequent pests and resulting diseases like malaria and schistosomiasis [15]. Like other parts of sub-Saharan Africa, most of the Ethiopian wetlands are at risk of habitat degradation and habitat loss due to population growth and other factors such as on-site and off-site management problems, the cultivation of wetlands, and the occurrence of drought [16]. Wetland bird species' diversity and abundance have been threatened due to various anthropogenic activities [9].

Understanding the state of the species can help with the management of ecosystems and the services they provide. Bird species have a significant functional role in wetlands [17]. The number of species present and how evenly the individuals are distributed among these species determine how diverse the bird species are [9]. Bird species diversity and assemblage vary with the seasons and types of habitat. According to Ali et al. [18], the number of species present during various seasons is the only difference between bird assemblages. They showed that wetland managers should be extremely concerned about the sharp loss in species diversity as well as the seasonal persistence of dominant assemblages. The movement of birds, the availability of food, the suitability of the habitat, a wetland's geo-physiological structure, and its size all affect diversity and distribution patterns [19].

The wetlands of the study area and their surroundings are a haven for several bird species, including the black-crowned crane (*Balearica pavonina*) and the thick-billed raven (*Corvus crassirostris*). There was no prior research carried out on the bird species inhabiting the two studied wetlands. This study will provide information on the diversity and abundance of wetland bird species in the study area, and it can serve as baseline information for other researchers who will be interested in filling the gap for sustainable conservation of the bird species. In addition to this, the study could be an input for future biodiversity conservation activities in the Hurri and Loga wetlands of Gomma Woreda, southwest Ethiopia. Therefore, the purpose of this study was to address the issues surrounding the variations in bird species diversity and assemblage structure between the dry and wet seasons in the two wetlands in southwest Ethiopia.

## Materials and methods

### The study area

**Fig. 1:**
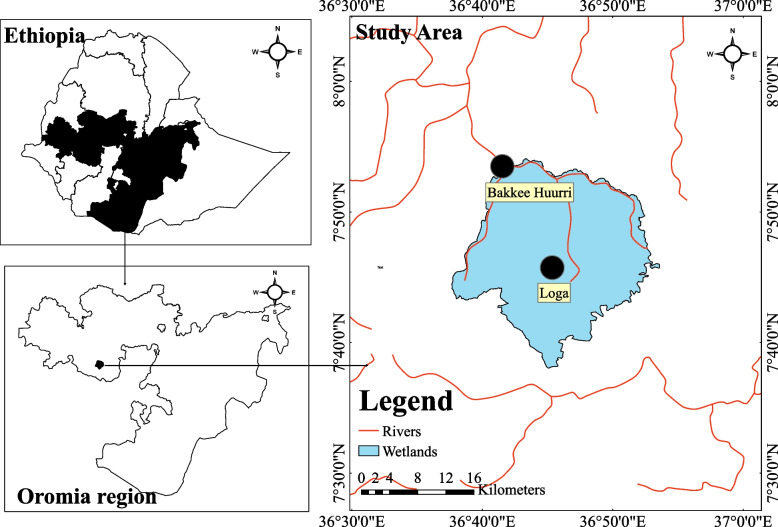
A study area map (Own source)

Hurri and Loga wetlands are located around Gomma woreda (equivalent to a district), Oromia Regional State. The elevation of Gomma woreda is 1636 m above sea level. The study area is located at 08° 43′ 00′′ to 07° 39′ 00′′ N latitude and 36° 22′ 00′′ to 36° 49′ 00′′ E longitude. The area has a total of 864.69 km^2^ of coverage (Fig. 1). It is bordered by Gumay and Gera Wereda to the west; Mana Woreda to the east; Limu Kosa Woreda and Buno Bedele Zone to the north; and Seka Chokesra to the south. Aggaro Town, which is the capital city of the woreda, is located 390 km from Addis Ababa on the way through Jimma.

Specifically, the two wetlands are found around Keta Muduga, which is the place where the foundation for coffee (*Coffea arabica*) production in Ethiopia was laid. Hurri wetland covers an area of 11.7 km^2^, while Loga is smaller (7 km^2^). The two wetlands have a total surface area of 18.7 km^2^. Hurri wetland is dominated by marshes and swamps with different water levels in different seasons, whereas the coastal area of Loga wetland is covered by different types of vegetation. The littoral areas of the wetlands are covered with grass species, including *Sporobolus pyramidalis* and *Hyparrhenia rufa*. Eucalyptus tree plantations and brick production were commonly observed practices around the Loga wetland habitat. However, livestock grazing and farming activities were the main activities in the Hurri wetland habitat. The average annual temperature in the area ranges between 10 °C and 30 °C, whereas the mean annual rainfall ranges between 0.3 mm and 233 cm [20].

### Methods

A reconnaissance survey was conducted for a week in January 2020 in order to become acquainted with the study area. Two different wetlands were purposefully selected for this study, namely the Hurri and Loga wetlands.

The point count method was used to study the bird assemblage structure and diversity in the study area [21, 22]. Data was recorded by distributing points in the given habitat and selecting points from the distributed points on a random basis. Two and one counting blocks were used for Hurri and Loga wetlands, respectively. For each counting block, six and four point counts were used for each wetland habitat in each season, respectively. With the help of a GPS (Garmin GPSMAP 64 s) and flagging tape, a total of 20 points were set up during both the dry and wet seasons. Locations in each habitat were at least 25 m away from the surrounding forest boundary. A laser meter was used to measure the distance and angle between the observer and the birds. At each location, activities such as bird diversity, abundance, and locations were recorded. A colored polygene sheet was used to mark each counted block. The radius of point-counting blocks was set at bands based on the bird’s detectability test during the reconnaissance survey [23, 24].

Data collection was carried out from January 2020 to July 2020, both during the dry (February to April) and wet (May to July) seasons, following the work of Amare [25]. Three trips were made to the research region each month. In order to reduce disturbance during point counts, a waiting interval of five minutes was utilized for transportation and bird adjustment before the count, and a further ten minutes were employed for bird observation [26, 27]. Species were visually and acoustically recognized within a 25-m radius using binoculars (8 × 30 and 8 × 40) and/or the human eye during each 10-min sample interval [27]. During point counts, certain audio recordings were later used for identification purposes. Birds flying overhead within the point radius were not counted [28]. The current sampling period's time and weather were noted during bird adjustment phases. Each point was 100 m away from the roadside to avoid the edge effect, and at each point count station, a minimum distance of 200 m to 300 m was maintained using the Global Positioning System to avoid double counting of the same individual species of birds [29]. Data collection was performed in the morning from 06:00 to 09:00 h and in the afternoon from 15:00 to 17:00 h, when the activity of birds becomes prominent [30]. Bird species names and populations were counted during the survey through direct observation. Using common bird field guides, the birds were identified and grouped into their appropriate taxonomic groups [31, 32]. For more assurance, a picture of the birds was also shot with a digital camera. The sounds of the birds were also used to identify them.

### Data analysis

All the recorded bird species were analysed using various parameters like the Shannon Index (H’) [33], species evenness (E), species abundance, and richness. The number of individuals recorded for each bird species was evaluated using the Species Diversity Index (H’). The values range between 0, indicating low community complexity, and 4 and above, indicating high community complexity.

- **Relative Density**
  $$H\mathrm{^{\prime}}=-\sum_{i=1}^{s}{p}_{i}\mathrm{ln}{p}_{i}$$

Where H’ = diversity index; Pi = the proportion of each species in the sample; and ln.

Pi is the natural logarithm of this proportion.

Abundance: Using the work of Bull [34], the abundance of bird species in the study area was computed by using the number of individual birds of particular species in the study area as a percentage of the total bird population of a given area.

$$\mathrm{Abundance}=\frac{\mathrm{the total number of individuals in all sampling units}}{\mathrm{total number of occurrence sampling units}}$$

$$\mathrm{Evenness}=\frac{\mathrm{H}}{{\mathrm{H}}_{max}}$$

Where, H =Shannons Diversity Index, and ${\mathrm{H}}_{max}$ =maximum diversity possible.

### Richness

The number of species per sample is a measure of richness. The more species present in the sample, the “richer” the sample becomes. Margalef’s index was used as a simple measure of species richness.

Margalef’s index = (S-1/In N).

where S is the total number of species, N is the total number of individuals in the sample, and In is the natural logarithm.

To evaluate bird species variation between habitats in the dry and wet seasons, a one-way ANOVA test was employed. The similarity among habitats and seasons in terms of bird species composition was evaluated using the Similarity Index (SI) = 2C/A + B [35].

Where SI denotes the similarity index, A denotes the number of species found in site A, B denotes the number of species found in site B, and C denotes the number of species found in both sites A and B. A sample correlation analysis of bird species was done using Pearson chi-square with a 5% significance level.

## Result

### Species diversity

**Fig. 2:**
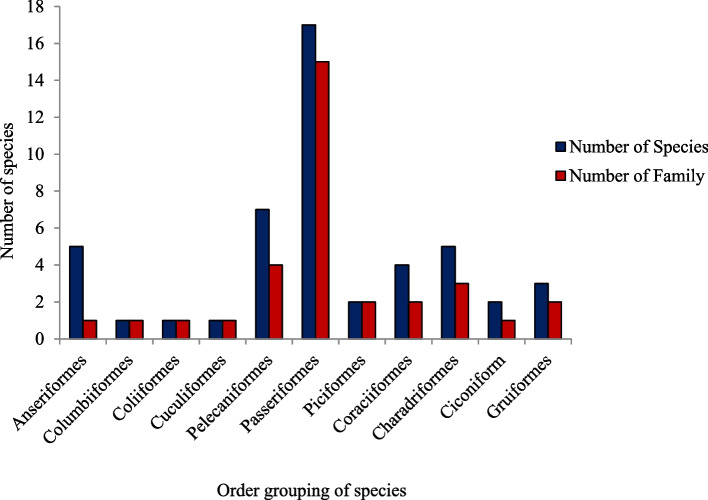
Bird orders and number of species in Hurri and Loga wetlands

During the study period, a total of 1769 individual birds, including 36 species, 11 orders, and 24 families, were recorded in the study area. In the two wetlands, a total of 1630 and 962 individual birds were recorded during the wet and dry seasons, respectively. During the wet and dry seasons, the Hurri wetland recorded 16 and 11 bird species, respectively, while the Loga wetland recorded 23 and 17 bird species, respectively. The Thick-billed Raven (*Corves crassirostris* Rüppell, 1836) and Banded Barbet (*Lybus undates* Rüppell, 1837) are endemic to Ethiopia, while nine pale arctic migrant species, 20 residents, and the remaining five species are partially migrant. Black Crowned Crane (*Balearica pavonina* Linnaeus, 1758) and Wattled Crane (*Grus carunculata* Gmelin, JF, 1789) were also observed in the study area (Tables 1 and 2). The order Passeriformes is represented by the highest number of species (*N* = 17), followed by the order Pelecaniformes (*N* = 7), orders Anseriformes and Charadriformes with five species each, Coraciiformes (*N* = 4), Gruiformes (*N* = 3), orders Piciformes and Ciconiformes with two species each, and orders Columbiformes, Cuculiformes, and Coliiformes with a single species each (Fig. 2). Order Passeriformes also had the highest number of families (*N* = 15), followed by order Pelecaniformes (*N* = 4), Charadriformes (*N* = 3), orders Piciformes, Coraciiformes, and Gruiformes with two families each, and orders Anseriformes, Columbiformes, Cuculiformes, Coliiformes, and Ciconiformes with a single family each (Fig. 2).

**Table 1:** The diversity of bird species in the study area during the wet and dry seasons

Wetland	Season	NS	NI	RI	H'	H'/Hmax
Hurri	Dry	11	218	1.9	2.283	0.9520
	Wet	16	416	2.5	2.643	0.9531
Loga	Dry	17	408	2.7	2.738	0.9665
	Wet	23	727	3.3	3.089	0.9853

*NS* Number of species, *NI* Number of individuals, *RI* Richness, *H'* Shannon–Weaver diversity index, *H'/H'max* Evenness, *H'max* ln(S)

**Table 2:** The bird species that were observed in the study area (number of individuals)

Order	Common name	IUCN Category	Scientific name	Family	Season	
					Dry	Wet
Anseriformes	Egyptian Goose	LC	*Alopochen aegyptiaca*	Anatidae	0	33
	White-backed Duck	LC	*Thalassomis leuconotus*	Anatidae	30	66
	White-faced Tree-Duck	LC	*Dendrocygna viduata*	Anatidae	0	35
	Fulvous Whistling-Duck	LC	*Dendrocygna bicolor*	Anatidae	0	24
	African Black Duck	LC	*Anas sparsa*	Anatidae	20	0
Columbiformes	Bruce’s Green-Pigeon	LC	*Treron waalia*	Columbidae	0	16
Coliiformes	Speckled Mousebird	LC	*Colius striatus*	Collidae	14	19
Cuculiformes	Blue-headed Coucal	LC	*Centropus monachus*	Cuculidae	0	18
Pelecaniformes	Cattle Egret	LC	*Bubulcus ibis*	Ardeidae	62	30
	Grey Heron	LC	*Ardea cinerea*	Ardeidae	0	15
	Hadada Ibis	LC	*Bostrychia hagedash*	Threskiornithidae	96	140
	Great White Pelican	LC	*Pelecanus onocrotalus*	Pelicanidae	19	16
	African Sacred Ibis	LC	*Threskiornis aethiopicus*	Threskiornithidae	56	71
	Royal Spoonbill	LC	*Platalea regia*	Threskiornithidae	0	36
	Hammercop	LC	*Scopus umbretta*	Scopidae	37	90
Passeriformes	Thick-billed Raven	LC	*Corves crassirostris*	Corvidae	0	27
	Parrot-billed Sparrow	LC	*Passer gongonensis*	Passeridae	34	33
	Ruppell’s Robin-Chat	LC	*Cossyphase mirufa*	Musicapidae	19	0
	Red-billed Oxpecker	LC	*Buphagus erithriorhynchus*	Buphagidae	23	26
	Red-winged Warbler	LC	*Prinia erythropterus*	Cisticolidae	0	20
	Northern Masked-Weaver	LC	*Ploceus taeniopterus*	Ploceidae	0	41
	Bronze Mannikin	LC	*Spermestes cucullata*	Estrildidae	63	40
	Beautiful Sunbird	LC	*Cinnyris pulchella*	Nectariniidae	16	0
	Barred Warbler	LC	*Curruca nisoria*	Sylvinidae	0	18
	Abyssinian Citril	LC	*Crithagra citrinelloides*	Fringillidae	36	35
	Collared Sunbird	LC	*Hedydipna collaris*	Nectariniidae	15	12
	Common Bulbul	LC	*Pycnonotus barbatus*	Pycnonotidae	76	77
	Fork–tailed Drongo	LC	*Dicrurus adisimiss*	Dicruridae	0	38
	Lesser Masked Weaver	LC	*Ploceus intermedius*	Ploeceidae	0	38
	Lesser Whitethroat	LC	*Curruca curruca*	Sylviidae	23	61
	Garden Warbler	LC	*Sylvia borin*	Sylviidae	27	34
	Little Weaver	LC	*Ploceus luteolus*	Ploeceidae	35	37
Piciformes	Greater Honeyguide	LC	*Indicator indicator*	Indicatoridae	19	0
	Banded Barbet	LC	*Lybius undatus*	Lybiidae	20	106
Coraciformes	Malachite Kingfisher	LC	*Corythornis cristatus*	Alcedinidae	22	16
	Pied Kingfisher	LC	*Ceryie rudis*	Alcedinidae	24	50
	Giant Kingfisher	LC	*Megaceryie maxima*	Alcedinidae	20	18
	Silver-checked Hornbill	LC	*Bycanistes brevis*	Buceratidae	11	0
Charadriformes	African Jacana	LC	*Actophilornis africanus*	Jacanidae	80	0
	Green-capped Eremomela	LC	*Eremomela scotops*	Cisticolidae	27	45
	Black-headed Gull	LC	*Chroicocephalus ridibundus*	Laridae	0	18
	Whiskered Tern	LC	*Chlidonias hybrida*	Laridae	0	33
	Grey-headed Gull	LC	*Chroicocephalus cirrocephalus*	Laridae	0	21
Ciconiformes	Marabou Stork	LC	*Leptoptilos crumenifer*	Ciconidae	0	89
	Saddle-billed Stork	LC	*Ephippiorhynchus senegalensis*	Ciconidae	0	12
Gruiformes	Black Crowned Crane	VU	*Balearica pavonina*	Gruidae	36	50
	Red-knobbed Coot	LC	*Fulica cristata*	Heliornithiday	0	26
	Wattled Crane	VU	*Grus carunculata*	Gruidae	2	0

*LC* Least concern, *VU* Vulnerable

In general, overall bird species diversity was higher during the wet season in both habitats. Loga wetland has higher species diversity in both wet (H' = 3.089) and dry (H' = 2.738) conditions. The Hurri habitat, on the other hand, has a lower species diversity in both wet (H' = 2.643) and dry (H' = 2.283) conditions. The species’ evenness (E) during the dry season was 0.9520 and 0.9665 for Hurri and Loga wetland habitats, respectively (Table 1).

### Similarity index

#### Bird species’ similarity between seasons

Simpson’s similarity index (SI) of the bird species in the two wetland habitats indicated that a higher (SI = 0.65) similarity of bird species between the wet and dry seasons was observed at the Loga wetland habitat when compared to the Hurri wetland (SI = 0.59) (Table 3).

**Table 3:** The overall similarity (SI) of bird species within habitats during the wet and dry seasons

Wetland	Wet	Dry	Common species	SI (Similarity Index)
Hurri	16	11	8	0.59
Loga	23	17	13	0.65

#### Bird species’ similarity between habitats

Bird species showed similarities between Hurri and Loga wetlands. During the wet season, bird species similarity was higher (SI = 0.50) between Hurri and Loga wetlands. Besides, the species similarity during the dry season was 0.31 (Tables 4 and 5).

**Table 4:** Comparison of the similarity of bird species' habitats during the wet and dry seasons

Wetland	Season					
	Wet			Dry		
	Species No	SI	%	Species No	SI	%
Hurri wetland with Loga	11	0.50	50	5	0.31	31

**Table 5:** One-way ANOVA test for bird species variation between habitats in the dry and wet seasons

Season	Habitat	Number	Mean value	SD	F	Sig
Wet	Hurri	16	0.1650	0.059	0.782	0.05
	Loga	23	0.1786	0.165		
	Total	39	0.1718	0.112		
Dry	Hurri	11	0.207	0.063	4.53	0.05
	Loga	17	0.161	0.050		
	Total	28	0.184	0.054		

#### Relative abundance

**Fig. 3:**
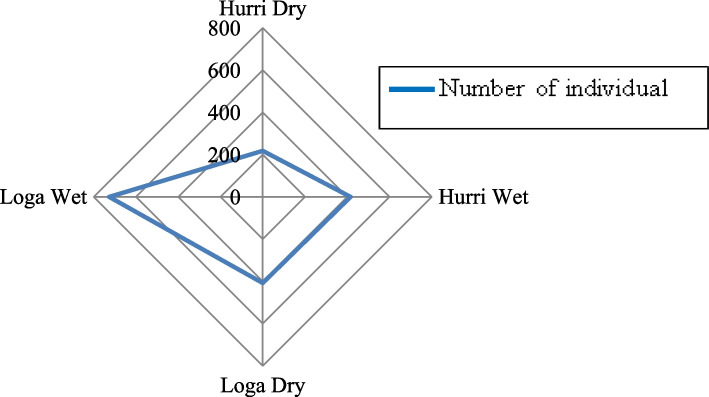
Individual (N) bird count in the study habitats

A total of 1135 and 634 individual birds were recorded from Loga and Hurri wetlands in both the wet and dry seasons. The higher number (*N* = 727) of individual birds was recorded from Loga wetland and the lower (*N* = 416) from Hurri during the wet season. Similarly, the higher (*N* = 408) and the lower (*N* = 218) numbers of individual birds were recorded from Loga and Hurri during the dry season, respectively. In general, in all habitats of the present study, bird species abundance during the wet season was high (Fig. 3).

There is a substantial variation in the data between the dry and wet seasons at Loga Wetland (mean = 24.00, SD = 10.03, *P* = 0.012). This demonstrated that the relative richness of bird species in the Loga wetland varies between the dry and wet seasons. However, there is no statistically significant difference in the relative abundance of bird species in the Hurri wetland environment between the dry and wet seasons. Additionally, the findings showed that while there is no difference in relative abundance between wetlands during the rainy season (F = 0.782; *P* > 0.05), there is a significant difference between wetlands during the dry season (F = 4.53; *P* < 0.05) (Table 6).

**Table 6:** Comparing the relative abundance of the wetland bird species in the dry and wet seasons

Season	Habitat	N	Mean value	SD	F	Sig
Dry	Loga	17	23.31	9.94	4.530	0.030*
	Hurri	11	19.81	9.70		
	Total	28	21.56	9.61		
Wet	Loga	23	30.29	12.85	0.782	0.463
	Hurri	16	25.75	12.91		
	Total	39	27.96	12.12		

## Discussion

A good indicator of conservation importance is species diversity, which is measured by the number of species and individuals present [36]. In the present study, a total of 36 bird species under 11 orders and 24 families were recorded from the two wetland areas. There was a difference in the number of individuals and species between the two wetlands during the dry and wet seasons. In the afro-tropical highland wetlands of the Awi zone and Wombera hotspot areas, Northwestern Ethiopia, 84 species and 23 families were recorded [37]. A total of 103 bird species belonging to 47 families and 14 orders were recorded in Lake Hawassa and parts of the Eastern Wetland habitats in southern Ethiopia during the wet and dry seasons [38]. The difference in the bird species composition between the wetlands may be due to the birds' nesting behavior, resource availability, and disturbance status. Changes in the condition of the habitat, disturbance, and resource access determine the diversity of the bird species in a particular environment [2, 13, 39–41]. Bird survival is endangered by the destruction of plant coverings, nesting and breeding habitats, and feeding grounds [42–44]. Wetland-specific bird species may go extinct locally in circumstances of severe wetland degradation and loss [45]. The Passeriformes order, one of the 11 orders, has the most species and families represented. With more than half of all known bird species in the world, the order Passeriformes is the biggest and most diversified group of avian organisms [46].

The abundance of birds in the two wetlands area revealed that the majority of species were abundant. This could be due to the greater detectability of birds in open wetland habitats as opposed to places with dense forest, which results in poor visibility. This is consistent with the findings of Amare and Girma [47] and Gibru and Biru [48]. This study indicated that the wetlands support a large number of bird species, including two endemic species, the Thick-billed Raven (*Corves crassirostris*) and the Banded Barbet (*Lybius undatus*). In addition to this, resident and migrant bird species occur in a significant number, which provides an indication that the area is a satisfactory habitat for resident bird species and a stopover for migrant bird species that can forage, loaf, rest, and refuel their energy. In a similar study that was carried out around Jimma town’s Boye Kitto and Kofe wetlands, 107 species of water birds were recorded [16]. The species composition of birds in different seasons was also determined for the study areas. In general, overall bird species diversity was highest during the wet season in all habitats. This might be due to the high species richness in this wet season.

According to Borgesio [49], wetland habitats provide many bird species with ample food resources such as frogs, worms, and insects. This study, however, found that among the two wetland habitats, the highest species diversity was recorded in the Loga wetland habitat. Further, the presence of a variety of vegetation around this wetland is probably a contributing factor. Smith [50] described how food resources are one of the key factors in determining the species diversity in a particular area. On the other hand, in the Hurri wetland habitat, relatively less bird diversity was observed. This might be due to more anthropogenic activities taking place around this wetland habitat. For this reason, birds do not get an adequate place for nesting and breeding. Meyer and Turner [51] described how the conversion of wetlands for agriculture and industrial ports affects the nesting and breeding sites of many bird species.

The result of species diversity analysis revealed that species composition is different among areas and months because of habitat differences, seasonal movement patterns, local and regional habitat changes, large-scale population changes, and climatic conditions [52]. The present study revealed that the seasonal occurrence of bird species in the two wetlands was different. In general, most bird species were locally common.

A total of 1626 individuals of 57 species of birds were observed during the wet season and 962 individuals of 41 species during the dry season in the two types of habitats of the study area (Table 2). The decline in global bird diversity has been linked to a number of anthropogenic factors, including pollution [53], water fluctuation [54, 55], habitat and landscape configurations, and the influence of the surrounding physiographic matrix [56]. The seasonal occurrence of bird species in the two wetlands was different. This difference might be due to the availability of food resources, habitat conditions, breeding season, and migratory behavior of bird species [57]. In a similar way, Gaston and Blackburn [58] explained that the distinct seasonality of rainfall and seasonal variation in the abundance of food resources resulted in seasonal changes in the abundance of birds. Furthermore, the temporal decoupling of food resources and bird numbers, variable climate harshness in different regions, or individuals' inability to reach isolated areas all have an impact on the migratory bird population [59].

In general, wetlands are important feeding and breeding areas for birds. Farmers around the wetlands cultivate the area during both the wet and dry seasons, with crops such as maize and sorghum becoming the dominant crops in the study areas. At present, the unusually high level of human encroachment has led to a reduction in the size of the wetlands, which has resulted in many areas being under permanent cultivation. Ultimately, this could eliminate the bird’s habitat unless concerned bodies are involved in conservation measures. To conserve the wetlands and the bird population of the study area, a management plan should be prepared emphasizing an avenue for the sustainable utilization of the resources of the wetland without jeopardizing its continued ecological values and function. As with all ecological studies, ours also had some shortcomings during data collection and analysis.

## Conclusion

The Hurri and Loga wetlands have a high bird species diversity since they are homes to a variety of bird species. The two vulnerable species (Wattled Crane and Black Crowned Crane) are two of the 36 species recognized, and they frequently rely on these wetlands. Therefore, to protect these species and the others, conserving and restoring the wetland has high ecological and economic value. This demonstrated the presence of significant populations, and the research area meets the ecological needs of the local bird species. Despite the fact that the wetlands are home to numerous bird populations, there are signs of anthropogenic activity nearby that warrant additional examination in order to learn more about the extent of these impacts and to plan the most effective conservation measures. The preservation of all biological diversity in general and bird species in particular should be a top priority. Accordingly, a management strategy that emphasizes the wetlands' long-term use ought to be established. Therefore, it is important to develop and implement community-based conservation initiatives as well as set up accountable institutions and wetland management policies in order to safeguard wetlands in the study area.

## Data Availability

All the data generated or analyzed during this study are included in this published article.
